# Implications of sampling design and sample size for national carbon accounting systems

**DOI:** 10.1186/1750-0680-6-10

**Published:** 2011-11-08

**Authors:** Michael Köhl, Andrew Lister, Charles T Scott, Thomas Baldauf, Daniel Plugge

**Affiliations:** 1 University of Hamburg, World Forestry, Leuschnerstr. 91, D-21031 Hamburg, Germany; 2 Institute for World Forestry, Johann Heinrich von Thünen-Institute (vTI), Leuschnerstr. 91, D-21031 Hamburg, Germany; 3 USDA Forest Service, Northeastern Research Station, 11 Campus Blvd, Suite 200, Newtown Square, PA, 19073, USA

## Abstract

**Background:**

Countries willing to adopt a REDD regime need to establish a national Measurement, Reporting and Verification (MRV) system that provides information on forest carbon stocks and carbon stock changes. Due to the extensive areas covered by forests the information is generally obtained by sample based surveys. Most operational sampling approaches utilize a combination of earth-observation data and in-situ field assessments as data sources.

**Results:**

We compared the cost-efficiency of four different sampling design alternatives (simple random sampling, regression estimators, stratified sampling, 2-phase sampling with regression estimators) that have been proposed in the scope of REDD. Three of the design alternatives provide for a combination of in-situ and earth-observation data. Under different settings of remote sensing coverage, cost per field plot, cost of remote sensing imagery, correlation between attributes quantified in remote sensing and field data, as well as population variability and the percent standard error over total survey cost was calculated. The cost-efficiency of forest carbon stock assessments is driven by the sampling design chosen. Our results indicate that the cost of remote sensing imagery is decisive for the cost-efficiency of a sampling design. The variability of the sample population impairs cost-efficiency, but does not reverse the pattern of cost-efficiency of the individual design alternatives.

**Conclusions, brief summary and potential implications:**

Our results clearly indicate that it is important to consider cost-efficiency in the development of forest carbon stock assessments and the selection of remote sensing techniques. The development of MRV-systems for REDD need to be based on a sound optimization process that compares different data sources and sampling designs with respect to their cost-efficiency. This helps to reduce the uncertainties related with the quantification of carbon stocks and to increase the financial benefits from adopting a REDD regime.

## Background

In the 1990's tropical deforestation was estimated to cause approximately 20 percent of the global anthropogenic carbon emissions [1]. Between 1997 and 2006, deforestation, forest degradation and peatland fires contributed between 8 and 20 percent to the global anthropogenic carbon emissions [2]. FAO [3] estimated an annual loss of carbon stocks in forest biomass of 0.5 Gt between 1990 and 2010, which is considered to be mainly a result of tropical deforestation. At their 16th meeting in Cancun in 2010, the Parties of the United Nations Framework Convention on Climate change (UNFCCC) approved the inclusion of a reduction of emissions from deforestation and forest degradation (REDD) mechanism as an eligible action to prevent climate changes and global warming in post-2012 commitment periods of the Kyoto Protocol (KP).

So far no financial value has been assigned to the carbon stored in forests. Decisions about future land use are driven by the potential income from alternative forms of land management rather than maintaining forests as non-disposable intangible assets. REDD introduces a new land use paradigm in which developed countries provide financial resources for incentives to developing countries to reduce carbon emissions from deforestation and forest degradation. Financial benefits are based on quantified carbon emission reductions relative to a pre-established reference level [4]. Due to the financial arrangements between developed and developing countries participating in a future REDD mechanism, there is a requirement for reliable and verifiable data on carbon emission reduction efforts [5]. Countries willing to adopt a REDD regime need to establish a national system for Measurement, Reporting and Verification (MRV) that provides information on forest carbon stock changes. While some authors see MRV systems as easy-to-apply tools [6], others describe the difficulties of implementation and operational applications [7-9].

The objective of this paper is to demonstrate the implications of sampling designs and sample sizes on the cost-efficiency of the measurement component of MRV systems. We chose four sampling approaches and anticipated different cost schemes for field surveys and remote sensing imagery to show the effect of both the inventory designs and the associated costs on the cost-efficiency and reliability of carbon inventory and monitoring systems. Assumptions and methods used in our study are compatible with those laid down in the IPCC GPG (Intergovernmental Panel on Climate Change Good Practice Guidance) [10].

Estimating forest carbon stock changes includes assessments of deforestation rates and associated carbon stock loss, afforestation and reforestation rates and associated carbon stock gains, and changes of carbon stocks in forests that remain forests. The approach presented in the IPCC GPG quantifies emissions or removals from carbon stocks within a given period as the product of the extent of human activity (activity data, AD) and the emissions-removals ratio per unit of activity (emission factor, EF). Information on AD and EF can be obtained in different ways, the most complex and reliable being from detailed, spatially dense forest monitoring and modeling data. The GPG classify the approaches in three categories (so called "Tiers") [11] with respect to requirements for data, analysis procedures, and reliability. Since continuous forest inventory data collected with a valid statistical sampling design allows for complex assessment and analysis procedures and results in reliable estimates with known (sampling) errors, they are assigned the highest Tier, i.e. Tier 3.

The IPCC Guidelines use six broad land-use categories to report emissions and removals from land use and land use conversions: forest land, cropland, grassland, wetlands, settlements, and other land. The six categories can be further subdivided on the national level to capture differences between climate, soil, ecological zones, and management practices [11]. In addition, IPCC defines five carbon pools which are to be considered for reporting carbon stock changes on forest land: aboveground and belowground biomass, dead wood, litter, and soil organic matter.

In forest inventories changes are generally assessed as the difference of an attribute (e.g. forest area, timber or biomass volume, stand age, timber value, carbon stock) between successive occasions [12-15]. This approach conforms to the so-called "stock difference method", which is along with the "the gain-loss method" presented by IPCC [11] to assess carbon stock changes.

From a statistical point of view two kinds of errors can occur when inference is drawn from monitoring data (Table 1). A Type 1 error would result if a change is inferred from the monitoring data though no change occurred in reality, while under a Type 2 error a real change would not be detected by monitoring. In the scope of REDD a Type 1 error could represent the risk of countries to report a change of carbon stock where the true carbon stocks did not change, while a Type 2 error would result in reporting no change while real carbon stocks decreased or increased. These types of errors could thus either cause countries to fail to report emissions reductions that would earn them benefits, or cause donors to erroneously acknowledge a country for seemingly successful reductions.

**Table 1:** Inferences from monitoring data and associates errors

		Inference	
		
		No change	Change
Actual state	No change	correct	False positive (Type 1 error, α)
	
	Change	False negative (Type 2 error, β)	correct

The reliability of results can be quantified by giving their precision, accuracy, mean square error or bias. These words are often used synonymously in colloquial speech, but they are deliberately contrasted in the context of sampling statistics. In the following, we show the definitions of precision, bias, mean square error, and accuracy.

### Precision

Precision refers to the size of deviations from the estimated mean, $\hat{\mu }$, obtained by repeated application of a sampling procedure. It is quantified by the standard error or confidence intervals. The precision of a statistical estimate can be increased by increasing the number of observations.

### Bias

Bias, B, is the difference between the estimated mean and the true mean, thus is directly related to the accuracy of an estimate, as $\text{B}=\hat{\mu }-\mu$. A problem in surveys is that the presence of bias, i.e. the lack of accuracy, is often not known.

### Mean square error

A useful measure of reliability is the mean square error (MSE). It combines the precision of an estimate with its squared bias. The MSE of an estimate is a useful criterion to compare a biased and an unbiased estimate. According to Cochran ([16], p. 15) the MSE is formally,

$$\begin{array}{c} MSE\left(\hat{\mu }\right)=E{\left(\hat{\mu }-\mu \right)}^{2}=E{\left[\left(\hat{\mu }-m\right)+\left(m-\mu \right)\right]}^{2}\\ =\left(\text{variance of}\;\hat{\mu }\right)+{\left(\text{bias}\right)}^{2} \end{array}$$

### Accuracy

Accuracy refers to the size of deviations from the true mean, μ. It relates directly to the MSE. When comparing two estimators, the one with the smaller MSE is said to be more accurate [17].

For unbiased estimates the MSE and the precision are asymptotically identical. As the concept of MSE and the underlying figures are often not intuitively understood by many stakeholders, the use of confidence intervals is suggested [16,18]. Confidence intervals give an estimated range of values, which is calculated from the sample data and which are likely to include the unknown population (true) value. Albeit the GPG and other publications suggest differently [10,11], confidence intervals account for precision only and do not address bias or other non-sampling errors. The selection of a confidence level (e.g., 95%) specifies the probability that the confidence interval calculated will include the true parameter value. In forestry applications, especially in research, a common choice is a 95%-confidence level, which says that in 95% of the time, if repeated samples are taken with the same methods, the confidence interval that is generated will contain the true parameter value [15-17].

Dawkins [19] introduced the lower bound of a confidence interval as a surrogate for the minimum quantity to be expected with a given probability. The lower bound of confidence intervals can serve as a proxy for the Reliable Minimum Estimate (RME) which the IPCC-Good Practice Guidance suggests for addressing uncertainties in the assessment of changes in soil carbon. In the context of afforestation and reforestation activities under the Clean Development Mechanism (CDM) [20,21] the RME as a conservative measure has already been reflected in several UNFCCC documents. Grassi et al. [22] propose using the principle of conservativeness in order to "*address the potential incompleteness and high uncertainties of REDD estimates*".

Confidence intervals and standard errors are strongly influenced by the variability of the target population. IPCC [10] presents the variability of above ground biomass stock for different forest formations. Inventory concepts need to take into account both the required precision and budget constraints, in order to come up with an optimal inventory design. Countries in a REDD-readiness or demonstration phase [23] need to pay special attention to the cost-efficiency of proposed REDD monitoring concepts. It is good practice to evaluate alternative sampling concepts under the criterion of cost efficiency [24]. However, in the vast number of publications on REDD monitoring schemes the aspect of inventory cost seems to have been neglected. An exception is Hardcastle and Baird [25], who present a cost assessment for measuring and monitoring forest carbon for 25 countries. The cost figures they present are indicative of the levels of funding that would be required to achieve reporting at different Tier-levels ignoring and including degradation.

### Sampling design alternatives

Different sampling design alternatives can be used in the scope of REDD monitoring. These sampling designs can employ in-situ (field plot) data, remote sensing-based data, or a combination of the two. Typically, a combination of remote sensing and in-situ assessments is utilized to assess AD and EF. Remote sensing data provide geo-referenced information for extensive areas, but no direct information on carbon stocks inside forests. Field assessments do not allow for spatially explicit mapping of activity data (and are thus generally excluded from being the sole source of MRV data), but do provide data on tree attributes that enable the calculation of biomass, carbon stocks and changes in them. Especially where airborne instead of space-borne sensors are used, it can be prohibitive to cover large areas with remote sensing imagery. Similarly, field data collection campaigns can be costly, especially in areas that are hard to access. Table 2 gives an overview of some alternative inventory concepts for REDD and the underlying sampling designs.

**Table 2:** Data sources and sampling designs for REDD monitoring

Data sources	Sampling design	**Estimation procedures for means and variances of the mean **(from [15])
Field plots	Simple random sampling	$\bar{\text{y}}\text{=}\frac{\sum_{\text{i}=1}^{\text{n}}{\text{y}}_{\text{i}}}{\text{n}};\text{v(}\bar{\text{y}}\text{)=}\frac{\sum_{\text{i}=1}^{\text{n}}{\left({\text{y}}_{\text{i}}-\bar{\text{y}}\right)}^{2}}{\text{n}(\text{n}-1)}$
		
	Systematic sampling	

Field plots & full coverage remote sensing imagery	Stratified sampling Example: field plots and Spot satellite imagery	${\bar{\text{y}}}_{\text{st}}=\sum_{\text{h}=\text{1}}^{\text{L}}\frac{{\text{A}}_{\text{h}}}{\text{A}}\frac{\sum_{\text{i}=\text{1}}^{{\text{n}}_{\text{h}}}{\text{y}}_{\text{hi}}}{{\text{n}}_{\text{h}}}$ $\text{v}\left({\bar{\text{y}}}_{\text{st}}\right)=\sum_{\text{h}=\text{1}}^{\text{L}}\frac{{{\left(\frac{{\text{A}}_{\text{h}}}{\text{A}}\right)}^{\text{2}}}^{\;}{\text{s}}_{\text{h}}^{\text{2}}}{{\text{n}}_{\text{h}}}$;
	
	Regression estimators Example: field plots and TerraSarX (radar)	${\bar{\text{y}}}_{\text{lr}}=\bar{\text{y}}+\text{b(}\bar{\text{X}}-\bar{\text{x}}\text{)}$ $\text{v}\left({\bar{\text{y}}}_{\text{lr}}\right)\approx \frac{\sum_{\text{i}=\text{1}}^{\text{n}}{\left[\left({\text{y}}_{\text{i}}-\bar{\text{y}}\right)-\text{b}\left({\text{x}}_{\text{i}}-\bar{\text{x}}\right)\right]}^{\text{2}}}{\text{n(n}-\text{2)}}$

Field plots & partial coverage remote sensing imagery	2-phase sampling for stratification (double sampling for stratification) Example: field plots & aerial photography	${\bar{\text{y}}}_{\text{ds}}=\overset{\text{L}}{\underset{\text{h}=1}{\sum }}\frac{{\text{n}}_{\text{h}}^{\prime }}{\text{n’}}{\bar{\text{y}}}_{\text{h}}=\overset{\text{L}}{\underset{\text{h}=1}{\sum }}{\text{w}}_{\text{h}}{\bar{\text{y}}}_{\text{h}}$ $v\left({\bar{\text{y}}}_{\text{ds}}\right)=\frac{N-1}{N}\overset{\text{L}}{\underset{\text{h = 1}}{\sum }}\left(\frac{{\text{n}}_{\text{h}}-1}{\text{n}\prime \text{ - 1}}-\frac{{\text{n}}_{\text{L}}^{\prime }-1}{\text{N}-1}\right)\frac{{\text{W}}_{\text{h}}^{\prime }-{\text{s}}_{\text{h}}^{2}}{\text{n}\prime }+\frac{\text{N - n}\prime }{\text{N(n}\prime -1\text{)}}\overset{\text{L}}{\underset{\text{h = 1}}{\sum }}{\text{W}}_{\text{h}\left({\bar{\text{y}}}_{\text{h}}-{\bar{\text{y}}}_{\text{ds}}\right)}$ where $s_{h}^{2}=\frac{1}{n_{h}^{\prime }}\overset{L}{\underset{h=1}{\sum }}{\left(y_{hi}-{\bar{y}}_{\text{h}}\right)}^{2}$
	
	2-phase sampling with regression estimators Example: field plots & LiDAR	${\bar{\text{y}}}_{\text{lr}}=\bar{\text{y}}+\text{b(}\bar{\text{x}}\text{'}-\bar{\text{x}}\text{)}$ v(y¯lr)=sy.x2[1n+(x¯'−x¯)2∑(x¯'−x¯)2]+sy2−sy.x2n'−sy2N with $s_{y.x}^{2}=\frac{1}{n-2}\left[\sum_{i=1}^{n}{\left(y_{i}-\bar{y}\right)}^{2}-b^{2}\sum_{i=1}^{n}{\left(x_{i}-\bar{x}\right)}^{2}\right]$

Where:

y_i _= observation on field plot i; x_i _= observation in phase-1 sampling unit i; n = sample size, n_h _= sample size in stratum h; b = regression coefficient; N = population size; w_h _= weight of stratum h = A_h_/A;

A = inventory area; A_h _= area in stratum h, h = 1, ..., L; s_h_^2 ^= variance of y in stratum h;

In forest surveys, ***simple random sampling (SRS) ***and, more commonly, ***systematic sampling***, are typically used. In SRS, sampling units are chosen randomly. In systematic sampling, they are arranged in a systematic pattern, usually on a square grid or other regular geometric network. The starting point of the geometric network of sampling units is generally the only element of randomization in systematic sampling. However, some, such as the US Forest Inventory and Analysis (FIA) program, randomize within each cell of a hexagonal tessellation of the study area [26]. Ranneby [27] and Matérn and Ranneby [28] studied exact approaches to calculate variances from systematic sampling in a forest inventory context, and determined that using SRS variance equations results in overestimates of sampling error. In forest surveys it is good practice to approximate the standard errors of systematic sampling designs by the SRS equations and accept the overestimation of the sampling errors.

Combined in-situ/earth observation sample designs use auxiliary information obtained by remote sensing and field sampling systems simultaneously. The earth observation data can consist of derived data, such as a classification of remote sensing data into land-use strata, or unprocessed reflectance data from optical, radar or LiDAR sensors. Variables of interest such as biomass or carbon stock are assessed on a small sample of field plots, and these data are combined with the more densely-sampled earth observation data using statistical estimation procedures in order to generate estimates.

The use of spatially continuous earth observation datasets generally leads to stratified sampling or regression sampling designs. ***Regression estimators ***relate an auxiliary variable, which is measured or known for all population elements, N, to a variable of interest, which is assessed on a sub-sample of size n. Regression estimators are applicable whenever the constraints for the application of linear regression are satisfied. In practical applications, the assumptions and constraints of linear regression such as sufficient data across the entire value range, or homoscedasticity, can easily be violated for small units.

In ***stratified sampling ***thematic classes are obtained by classifying the remote sensing imagery and assigning the individual pixels (or polygons) into a fixed number of groups (strata). Thus, the idea of stratified sampling is to divide the population of N units into non-overlapping subpopulations of N1, N2, ..., NL units. The subpopulations are called strata. The strata are constructed to minimize the variance within strata, thus maximizing the differences between strata means. The characteristics of classes suitable for stratification do not necessarily reflect thematic information that is suitable for map production. In many cases combinations of thematic maps (i.e., different classification schemes) or totally artificial (i.e., thematically "meaningless") classes prove best for stratification purposes [15]. The n samples can be assigned to the strata equally, proportional (i.e., in proportion to strata sizes), optimal (i.e., by strata sizes and strata variances), or by Neyman allocation that in addition to strata size and variance includes the assessment costs per stratum [16]. For monitoring purposes proportional allocation proves most feasible, because changes in stratum assignments over time do not affect the probabilities of selection, thus complicating estimation [29,30].

In extensive surveys of large areas it is sometimes not possible to acquire full-coverage remote sensing imagery. That holds especially true when airborne instead of space-borne remote sensing data are to be used. Here, two-phase sampling designs offer an alternative by sampling both the variable of interest as well as the auxiliary variable. Stratified sampling and regression estimators can be applied as two-phase sampling for stratification and two-phase sampling with regression estimators.

In ***two-phase sampling with regression estimators ***the auxiliary variable x_i _is measured on a sub-sample of N. In this first phase a large sample of size n' is selected. In the second phase a random subsample of n' is selected where both x_i _and y_i _are measured and related via regression models. Two-phase sampling with regression estimation results in specific problems when used in practical applications. Among those problems are the need for calculating regression estimates for any variable considered, the assumptions for regression may be violated, no additive tables are obtained (table cells and margins are modeled separately), or not being able to analyze data on nominal and ordinal scales [31].

***Two-phase sampling for stratification ***is similar to stratified sampling - the difference being that the strata sizes are not measured but estimated by the large first phase sample. The variance $\text{v(}{\bar{\text{y}}}_{\text{ds}}\text{)}$ combines the within and between strata variation. For large N, $\text{v(}{\bar{\text{y}}}_{\text{st}}\text{)}$ can be used as an approximation for $\text{v(}{\bar{\text{y}}}_{\text{ds}}\text{)}$.

MRV systems need to provide figures on total rather than on mean carbon stocks and their respective changes. Therefore the equations presented in Table 2 need to be extended to total values. The population total and its variance is estimated from any mean by

$$\hat{\text{Y}}=\text{N}\bar{\text{y}}$$

$$\text{v(}\hat{\text{Y}}\text{)}={\text{N}}^{\text{2}}\text{v}\left(\bar{\text{y}}\right)$$

When $\bar{\text{y}}$ is related to a unit area (e.g. ha) then the population size N can be replaced by the area of the entire population A. Under the assumption that the estimates of means are normally distributed, the lower and upper confidence limits for the population mean and total are as follows:

$$\begin{array}{cc} {\bar{\text{y}}}_{\text{L}}=\bar{\text{y}}-\frac{\text{ts}}{\sqrt{\text{n}}}, & {\bar{\text{y}}}_{\text{U}}=\bar{\text{y}}+\frac{\text{ts}}{\sqrt{\text{n}}} \end{array}$$

$$\begin{array}{cc} {\hat{\text{Y}}}_{\text{L}}=\text{N}\bar{\text{y}}-\frac{\text{tNs}}{\sqrt{\text{n}}}, & {\hat{\text{Y}}}_{\text{U}}=\text{N}\bar{\text{y}}+\frac{\text{tNs}}{\sqrt{\text{n}}} \end{array}$$

For sample sizes that are sufficiently large (n > 60), the Student's t-value corresponds to the value of the normal deviate with the desired probability, e.g., t = 1.96 for 95% confidence levels with large sample sizes.

### Selecting the optimal design

Many inventory concepts have been presented for monitoring carbon stocks and carbon stock changes in the scope of REDD. Irrespective of the objective of a survey alternative, inventory concepts exist to choose from, including the utilized data sources (field assessments, remote sensing, maps etc.), the design of the sampling units (plot configuration), sampling rules and sample sizes. The potential design alternatives are influenced by a variety of factors such as the variability of the target population, budget allowance, or availability of auxiliary data sources and information (e.g. maps, remote sensing imagery, biomass models). A rational decision about the optimal design can be made only by comparing the set of alternatives under objective selection criteria that combine information on survey cost and the achievable reliability of the results. This allows for selecting the most cost-efficient design that either provides the best reliability under a given budget or provides the desired reliability by least cost. Discussions on survey design alternatives that lack the inclusion of cost are not very helpful for developing operational MRV-systems under a national REDD regime.

### Survey Costs

Survey costs are made up of fixed and variable cost components. Fixed costs are those that do not vary with sample sizes and design alternatives, but are common to all alternatives, for example cost for administration or research. As fixed costs are design independent they are not to be considered in the optimization process [24,32]. Design dependent costs include additional fixed costs for specific design alternatives and variable costs. Costs for visiting and measuring field samples are a typical example of variable costs, which are proportional to the number of field samples assessed. For stratified sampling, additional costs include acquisition, enhancement, and classification of remote sensing data as well as validation of the classification results.

Hardcastle and Baird [25] studied the readiness of 25 tropical countries for monitoring forests and reporting on REDD. For each country cost estimates are provided for implementing REDD MRV systems, the major drivers of costs being forest extent, stratification, and the appropriate choice of estimation method (Tier). They present the initial and recurrent cost separately for 4 alternatives:

1. Tier 2, Approach A: an accurate land-cover map is available, 300 sample plots are assessed in-situ, all carbon measurements are performed once at the beginning of the programme, future monitoring is focused on the assessment of human activities (activity data, AD) such as area changes by remote sensing data and requires only minimal field work.

2. Tier 2, Approach B: no accurate land-cover map is available, in-situ assessments are performed when activity monitoring by remote sensing identifies locations under change, the in-situ sampling intensity is considerably lower than under Tier 2, Approach A.

3. Tier 3, ignoring degradation: AD and emissions per unit of the activity (emission factors, EF) are assessed as under alternative 1 (Tier 2 Approach A), but remeasurements are made in permanent in-situ sample plots (about 1/3 of the original sample locations)

4. Tier 3, including degradation: alternative 3 is enhanced by further stratification of forests into the two classes "intact forests" and "non-intact forests", the number of field plots is moderately increased

The inventory concepts applied by Hardcastle and Baird [25] are generic rather than case-specific, as they do not result from an optimization process on the individual national levels. However, they are used for an approximate comparison of cost required to implement an operational REDD MRV scheme on the national level. Hardcastle and Baird [25] present respective costs for four alternatives over forest area. The cost per unit area decrease with increasing forest area, as the share of fixed costs in total costs decreases.

### Variability of the target population

Sample sizes and thus survey costs are directly linked to the variability of the sample population. Variability data for a population can be obtained by prior knowledge or by a pilot survey. For each variance component that is included in the estimation procedures, variability figures have to be specified. For stratified sampling this means specifying the variance by stratum for each key attribute of interest.

### Optimization

For each sampling alternative there exists an optimum combination of sample sizes. These optimum combinations should be used to compare the various design alternatives. In the optimization process variance functions and cost functions have to be linked in order to derive the optimal (i.e. most cost-efficient) sampling alternative. The optimum sampling design can be defined in two ways:

1. minimizing cost for a specified level of precision, or

2. minimizing variance for a specified cost.

In either case, the optimization requires that the cost and precision be expressed in terms of the sampling design and sample sizes.

## Results and Discussion

**Figure 1:**
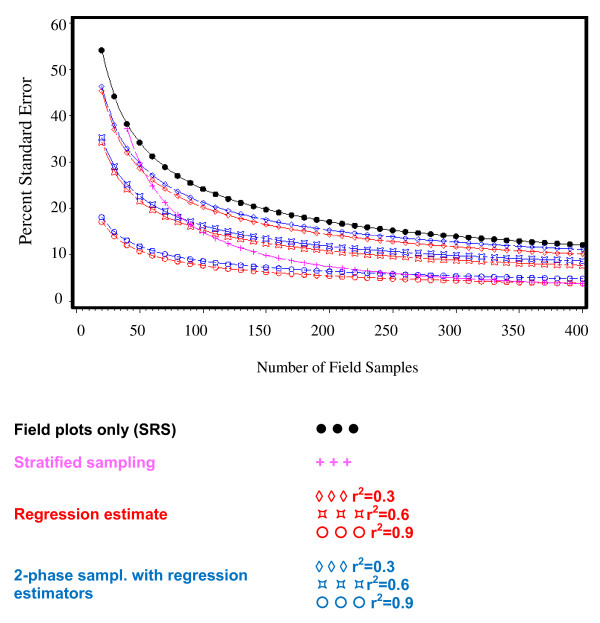
**Percent standard error over number of field samples (phase-1 coverage = 1%)**.

The results shown below were obtained based on the assumptions presented above utilizing the Puerto Rico dataset [33]. The percent sampling error of each of the simulated design alternatives is presented in Figure 1. As expected for each design alternative standard errors decrease with increasing sample sizes. The design alternative that used only field plots (SRS) and not any remote sensing derived auxiliary information consistently resulted in the largest percent standard errors.

From Figure 1 it can be seen that r^2^-values have a pronounced effect on standard errors. An increase of r^2 ^from 0.3 to 0.9 reduces the percent standard error by approximately 50 percent. The functional pattern of sample size and percent standard error is similar for all design alternatives except stratified sampling; under stratified sampling the gain in precision with increasing sample size is more pronounced. Under any sampling design the relative gain in efficiency decreases with increasing sample sizes. For our example there exists a drop-off point at a sample size of around n = 200, after which the percent standard error drop would not account for the increased cost to collect additional samples.

**Figure 2:**
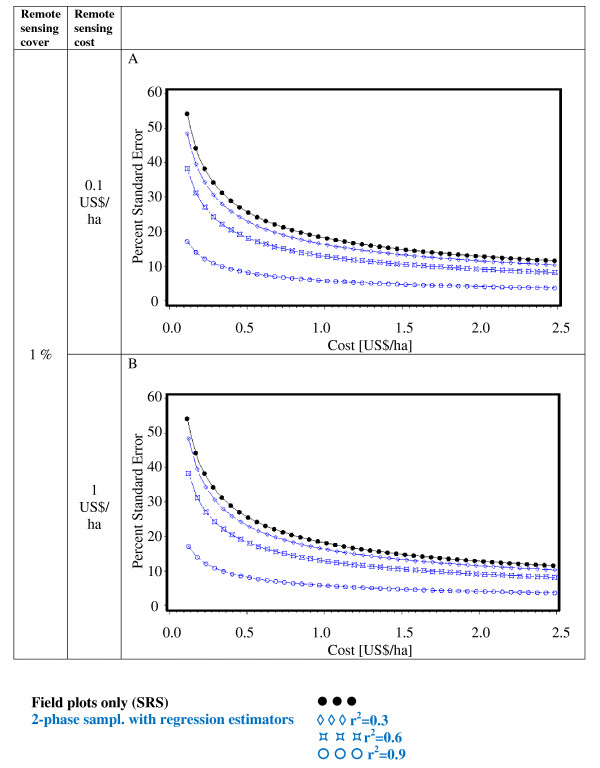
**Sampling design alternatives, field assessment cost: 5, 000 US$ per field plot, and remote sensing cover of 1%**.

**Figure 3:**
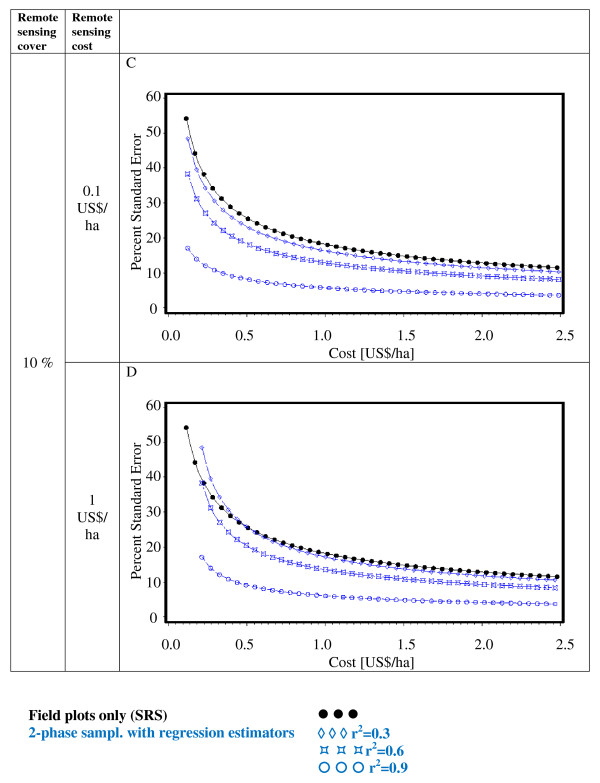
**Sampling design alternatives, field assessment cost: 5, 000 US$ per field plot, and remote sensing cover of 10%**.

**Figure 4:**
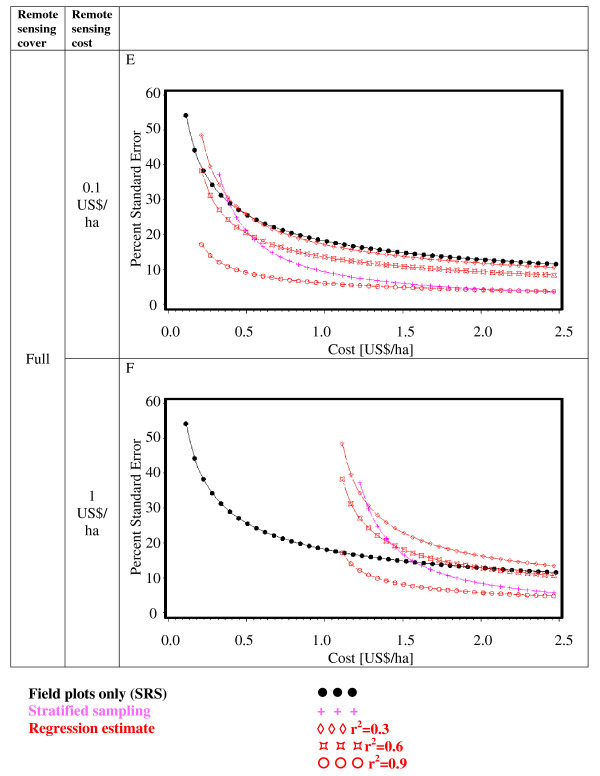
**Sampling design alternatives, field assessment cost: 5, 000 US$ per field plot, and full remote sensing cover**.

**Figure 5:**
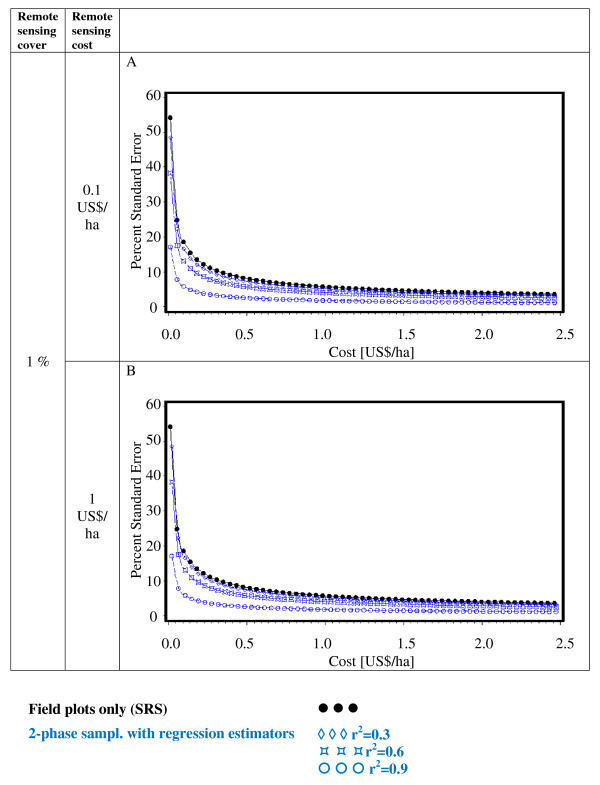
**Sampling design alternatives, field assessment cost: 500 US$ per field plot, and remote sensing cover of 1%**.

**Figure 6:**
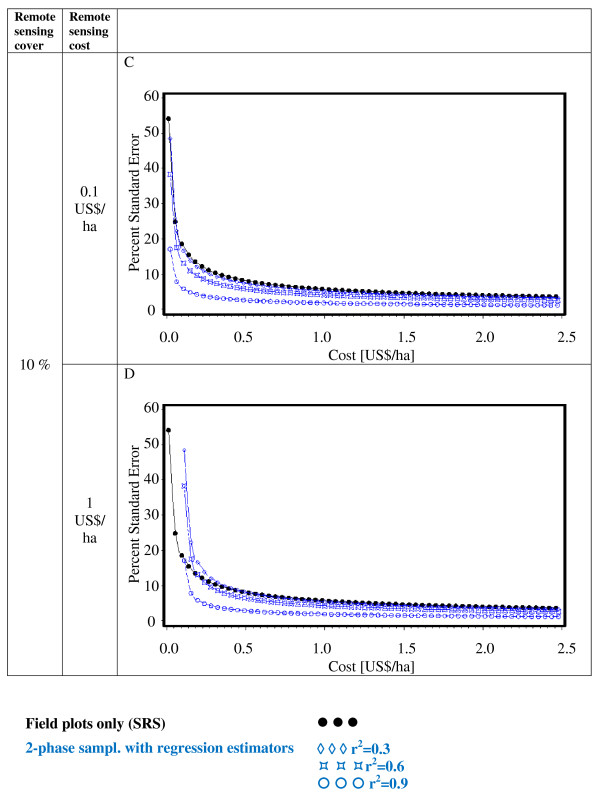
**Sampling design alternatives, field assessment cost: 500 US$ per field plot, and remote sensing cover of 10%**.

**Figure 7:**
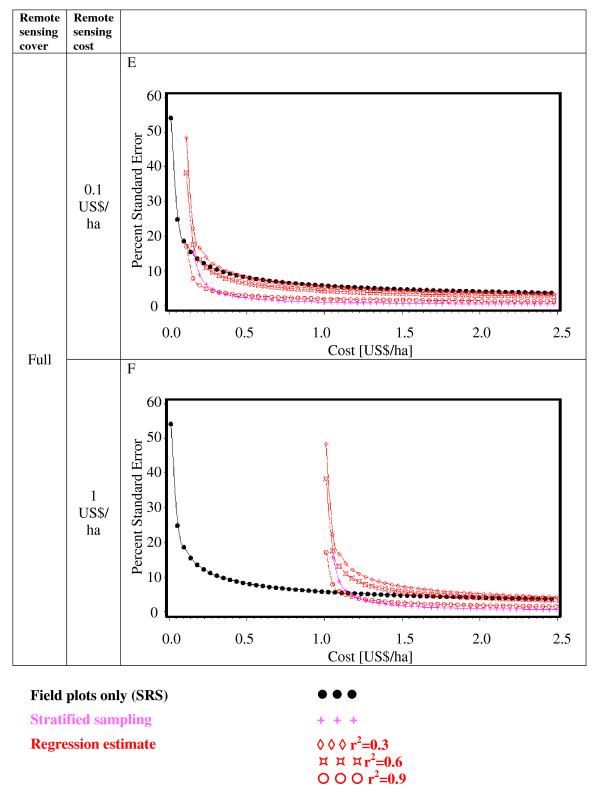
**Sampling design alternatives, field assessment cost: 500 US$ per field plot, and full remote sensing cover**.

Figures 2, 3, 4, 5, 6 and 7 present the percent standard error over cost and thus allow for the assessment of the cost-efficiency of the design alternatives. Four different scenarios are shown in these figures, which are a combination of cost of remote sensing imagery (0.1 US$/ha and 1 US$/ha) and phase 1 coverage (1 percent and 10 percent). The cost per field plot are set to 5, 000 US$ (Figures 2, 3 and 4) and 500 US$ (Figures 5, 6 and 7).

The design alternatives show similar behavior - rising cost reduces via the increased number of field plots assessed the percent standard error. The impact of r^2^-values as seen in Figure 1 can be translated into cost: for the same cost a r^2^-value of 0.9 reduces the percent standard error by half compared to an r^2^-value of 0.3. The gain in standard error per cost unit decreases with increasing cost until it reaches a more or less steady state.

For low costs of remote sensing as opposed to per field plot cost (Figure 2A, Figure 3C, Figure 5A,Figure 6C) the design alternatives utilizing remote sensing perform better than SRS, with the exception of stratified sampling for costs below 0.5 US$/ha and regression estimators with r^2 ^= 0.3 for costs below 0.4 US$/ha (Figure 4E, Figure 7E). The pattern of gain in percent standard error over cost is similar for all design alternatives except stratified sampling. Here the rate of reduction in sampling error is greater than the other alternatives, although there are higher initial costs (Figure 4EF, Figure 7EF). This makes stratified sampling the least cost-efficient alternative for low costs (fewer field plots) and the most cost-efficient for high costs (more field plots).

When the cost of remote sensing imagery is assumed to be 1 US$ per hectare, the design alternatives requiring full coverage of the auxiliary variable (regression estimation and stratification) (Figure 4F, Figure 7F) differ considerably in cost-efficiency from the 2-phase designs (Figure 2B, Figure 3D, Figure 5B, Figure 6D), which require only partial coverage. Stratification and regression estimators with r^2^-values of 0.6 reach the percent standard errors of the other designs at a cost level of 1.6 US$/ha, while regression estimation with an r^2^-value of 0.3 fail to achieve parity with the other designs until they reach a much higher cost-level. Where remote sensing information is costly, design alternatives utilizing only field plots or partial coverage with remote sensing imagery can thus be the more cost-efficient alternatives.

Figures 5, 6 and 7 show results of the sample design alternative comparison when field plots costs are assumed to be 500 US$/plot. In this case, cost efficiency is necessarily consistently better than for more expensive per plot costs (Figures 2, 3 and 4). The differences between design alternatives are less pronounced with low-cost remote sensing data; here differences in cost-efficiency between regression estimators and 2-phase sampling with regression estimators become negligible when cost are 0.3 US$/ha or higher (Figure 5A, Figure 6C, Figure 7E). Where the cost of remote sensing are higher (Figure 5B, Figure 6D, Figure 7F) full-coverage design alternatives are competitive only for higher total per hectare cost. Stratification and regression estimates with low r^2^-values are less efficient than SRS for moderate costs.

When remote sensing costs are assumed to be 1 US$/ha, stratified sampling and regression estimators can no longer compete with the other design alternatives (Figure 4F, Figure 7F). For a remote sensing coverage of 1 percent of the study area, 2-phase sampling with regression estimators is consistently more cost-efficient than SRS (Figure 2B, Figure 5B), while for a 10-percent remote sensing coverage this holds true only for r^2^-values of 0.9 (Figure 3D, Figure 6D).

**Figure 8:**
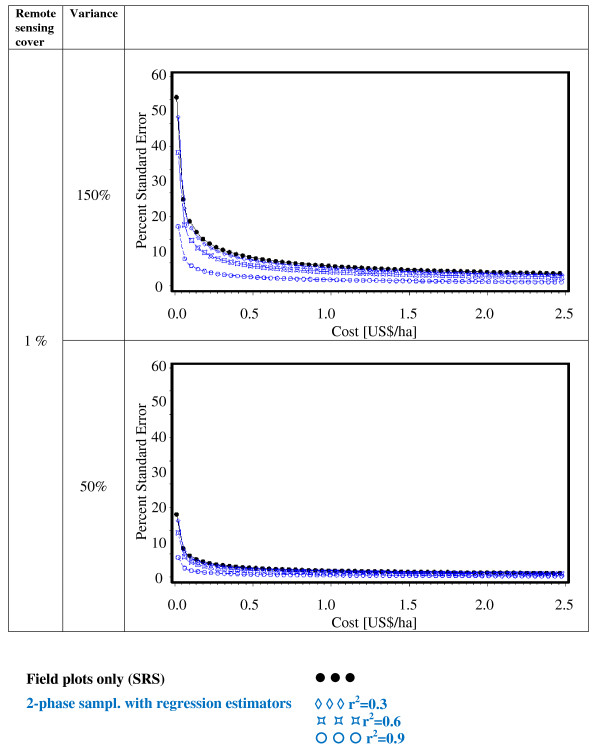
**Cost-efficiency under changing population variances (cost per field plot = 500 US$), and remote sensing cover of 1%**.

**Figure 9:**
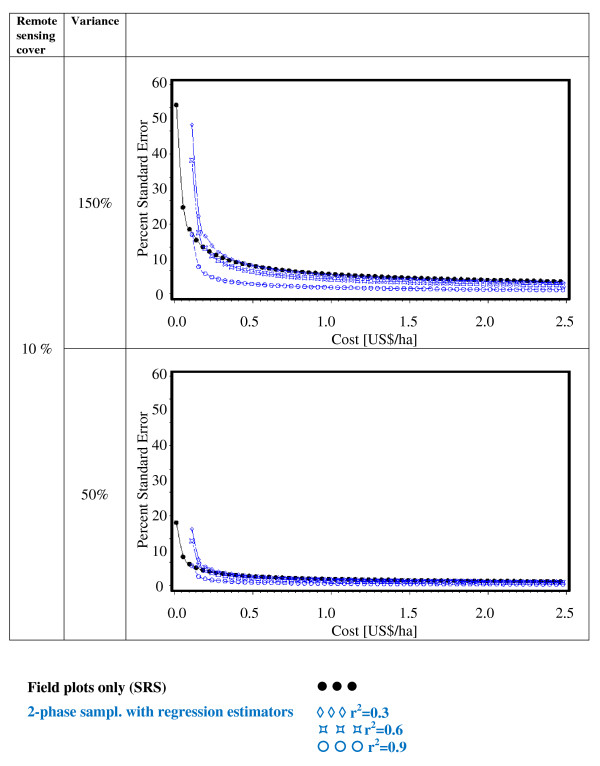
**Cost-efficiency under changing population variances (cost per field plot = 500 US$), and remote sensing cover of 10%**.

**Figure 10:**
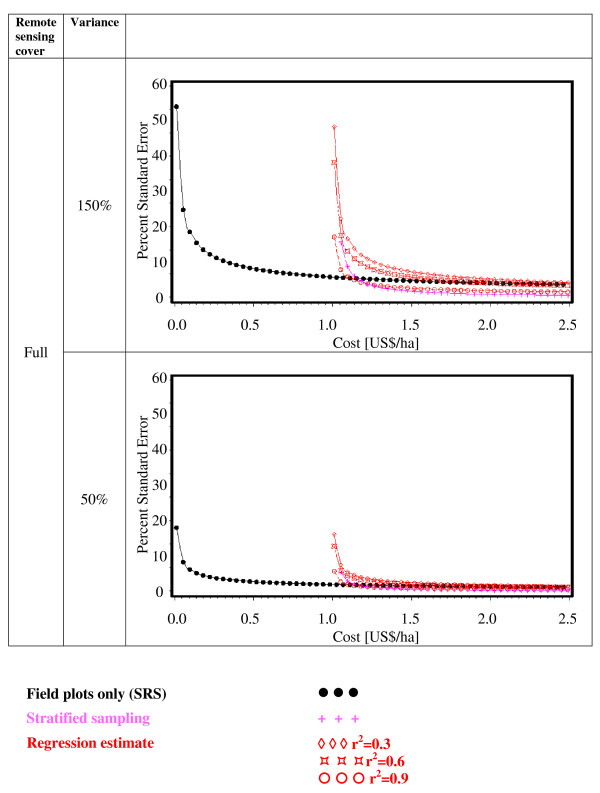
**Cost-efficiency under changing population variances (cost per field plot = 500 US$), and full remote sensing cover**.

While in Figures 2, 3, 4, 5, 6 and 7 constant cost for remote sensing imagery was assumed regardless of the type of coverage attained, Figures 8, 9 and 10 show a cost scenario that is more realistic for remote sensing applications. It is assumed that the cost for remote sensing imagery is higher when used for partial coverage than for wall-to-wall coverage. This is a typical situation when inventory approaches utilizing airborne LiDAR data are compared with those that use space-borne multispectral or RADAR data. In the scenarios presented in Figures 8, 9 and 10, the cost for full coverage remote sensing data was set to 0.01 US$/ha and to 1 US$/ha for partial coverage. Under these assumptions regression estimates that require full coverage are now more cost efficient than 2-phase sampling with regression estimators. For cost over 0.3 US$/ha stratified sampling becomes the most cost-efficient alternative.

From the equations given in Table 2 it is intuitively clear that changes in population variances affect standard errors but do not change the pattern of cost-efficiency. To illustrate this obvious matter of fact we simulated cost efficiency under different variance assumptions. The variances presented in Tab. 4 were by 50% inflated and decreased. The effects on cost-efficiency can be seen in Figures 8, 9 and 10. The absolute standard errors change, but the general pattern of the cost-efficiency curves is maintained. Similarly the relative order of the designing alternatives with respect to cost-efficiency does not change.

## Conclusions

In our simulation study we compared different sampling design alternatives in the scope of REDD and linked information on sampling variance with information on cost. This allowed us to characterize the effect of sampling design alternatives and sample sizes on the cost-efficiency of a REDD MRV- system. This approach facilitates the selection of the optimal design alternative for specific populations and monitoring objectives.

The optimization process offers a set of potential starting points for improvement. Sampling intensity, field plot design and sample design (including the potential for use of a remote sensing product as auxiliary data) are the most important control variables for developing a cost-efficient inventory and monitoring methodology. Given the assumptions we chose to adopt, our cost analysis study revealed that incorporating expensive (i.e. airborne) remote sensing data into the sample design for a forest carbon measurement survey can unnecessarily inflate the costs compared to other alternatives.

The results indicate that it is important to include cost-efficiency aspects in the selection of the remote sensing alternative to be used. It needs special justification if expensive remote sensing alternatives are suggested. Either they improve cost-efficiency or there are additional benefits beyond the mere estimation of carbon stock changes.

The development of MRV-systems for REDD needs to be based on a sound optimization function, where either costs are minimized for a desired level of precision, or variances are minimized for a specified budget. Design optimization has to consider the marginal benefit for improving the cost-efficiency. Increasing the budget for an assessment results in substantial improvements of standard errors in the beginning, but the marginal benefits become negligible for high costs. The definition of the ideal turning point is such essential for the design optimization. The turning point could be selected by applying the principles of capital budgeting or by expert opinion.

Monitoring cost are especially important in the context of REDD, as an MRV-system can be seen as an investment that aims to generate financial benefits. The amount of investment and the resulting reliability of the estimated carbon stock drives the financial gains, and thus rules the success of a REDD regime. This holds especially true in situations where deforestation is driven by the expectation of financial profits due to land-use change.

Uncertainty is a major issue in MRV-systems. Given the decreasing marginal benefit with increasing budgets indicates that increasing the sampling intensity is not the ultimate solution to improve the reliability of estimates. The application of models and functions renders necessary to transfer data assessments into estimates of carbon stock changes. The uncertainty underlying those models and functions has widely been discussed and was recognized by IPCC [11]. In relation to design optimization it could be a better choice to accept lower sampling intensities and resulting higher standard errors and invest into the improvement of models and functions. Extending the cost considerations from the cost-efficiency of sampling to the overall cost of a MRV-system turns design optimization into a process that is part of the entire desire to reduce uncertainties and make estimates of carbon stock changes more reliable.

## Materials and methods

Designing a monitoring system renders decisions on data sources, sample sizes, and sampling designs necessary, which in turn control inventory cost and cost-efficiency. To represent the interrelations between these inventory design components in a general and transferable way, we chose a simulation study approach. The simulation study was designed to repeatedly generate estimates on sampling errors with different combinations of design alternatives, samples sizes and costs. By analyzing the results of the simulation runs we hope to indentify principles that can help to guide design choices for REDD monitoring.

True population data on variance structures were taken from the Third Forest Inventory of Puerto Rico [33,34], which covers a total land area of 886, 996 ha. The forest life zones found on the mainland of Puerto Rico are "subtropical dry forest, subtropical moist forest, subtropical wet forest, subtropical rain forest, subtropical lower montane wet forest, and subtropical lower montane rain forest" [34], while on the islands of Vieques and Culebra subtropical dry forest conditions prevail. Field data were collected by FIA. Each FIA plots consists of four circular 14.6 m diameter subplots, with one subplot located in the center and three equidistant subplots distributed symmetrically around and located 31.6 m from the center subplot. The subplots occupy 0.07 ha, and the subplot array can be subtended by a circle of 0.4 ha in area [35,36].

Per plot aboveground biomass (AGB) figures were taken from the FIA data set. FIA estimates AGB by regression models that are either developed by the FIA program or compiled from the literature. The models predict aboveground biomass from individual tree dbh and total height measurements and provide the total oven-dry biomass in kilograms of all live aboveground tree parts, including stem, stump, branches, bark, seeds, and foliage. Carbon is calculated by multiplying estimated total biomass of all trees with dbh ≥ 2.5 cm by a factor of 0.5 [34]. Per plot values were expanded to unit area (hectare).

Table 3 provides the summary statistics of the data used for the case study for all observed plots and for the key forest types. A total of 956 plots were available of which 288 plots (or 30 percent) are located on forested areas and 678 plots on non-forest land. Both forested and non-forested plots were used in the simulation runs. For the entity of all plots a coefficient of variation of 242 percent was calculated, ranging from 40 percent in lower wet and rain forests to 137 percent in Mangrove forests.

**Table 3:** Summary statistics for above ground biomass*

Forest Type	N	Mean	Variance	Coeff. of Variation	Std Error
Mangrove	3	45.99	3, 949.77	136.64	36.28

Dry forest	54	54.17	2, 451.60	91.40	6.74

Moist forest	141	136.33	19, 248.57	101.77	11.68

Wet and rain forest	81	200.58	27, 611.02	82.84	18.46

Lower montane wet and rain forest	4	220.36	7, 675.41	39.76	43.80

Mixed	5	68.55	2, 021.61	65.59	20.11

All (288 forest plots; 678 non-forest plots)	956	41.59	10, 137.60	242.11	3.26

* AGB expanded to unit area (hectare)

The simulation study aims at comparing the efficiency in terms of percent sampling error with the underlying assessment cost and providing information on the cost-efficiency of different design alternatives. Four different sampling designs were selected for the simulation study:

- Simple random sampling (SRS); this alternative would represent a solely field-based assessment

- Regression estimators; under this alternative auxiliary data (e.g. LiDAR or RADAR backscatter) are assessed on a wall-to-wall coverage of remote sensing imagery and linked via regression estimates to the variable of interest (e.g., AGB) that is assessed on a (small) sub-sample of field plots.

- Stratified sampling; a wall-to-wall coverage of remote sensing imagery is utilized to separate the entire population into homogeneous strata. In each stratum field plots are assessed. The classification of multi-spectral, optical remote sensing data would be a common procedure to obtain the stratification of the inventory area.

- 2-phase sampling with regression estimators; the alternative is similar to regression estimators, but requires only a partial coverage of the inventory area by remote sensing imagery. Where airborne remote sensing systems such as LiDAR render data assessment on flight lines rather than full coverage necessary, this sampling alternative is the preferred method. For the simulations study we used a phase-1 coverage of 1 percent and 10 percent of the entire inventory area.

Simple random sample will serve as the baseline for comparing alternative sampling designs. The performance of both, regression estimators and 2-phase sampling with regression estimators depends on the correlation between the auxiliary variable and the variable of interest. Drake et al. [37] used metrics from large-footprint LiDAR and modeled plot-level biomass with r^2 ^= 0.93 for a 1, 536 ha area in Costa Rica stocked by primary and secondary wet tropical forest, abandoned pasture and plantations, and agro-forestry. Even higher r^2^-values could be found in boreal and temperate mono-species forests. For example, Means et al. [38] found on 26 plots (approx. 6.5 ha) primarily of Douglas-fir and western hemlock r^2 ^values of 0.96 for the estimation of AGB. Considerably lower r^2^-values were found for volume (0.66) and biomass (0.59) by van Aardt et al. [39], who used small-footprint LiDAR to study a LiDAR-based, object-oriented approach to forest volume and aboveground biomass modeling in temperate forests. We included r^2^-values of 0.9, 0.6, and 0.3 in our simulation study to extent the informative value to operational applications and to show the effect of the underlying correlation between auxiliary and field data on the cost-efficiency of the design.

In order to prepare the data for the simulation of stratified sampling, the Jenks Natural Breaks Classification method was applied [40]. Jenk's optimization method assigns values to a given number of classes with the objective of minimizing variances within classes while maximizing between class means (Table 4). In terms of the simulation study, this results in an optimal stratification rule; not any remote sensing technology could perform better.

**Table 4:** Stratification by Jenk's Natural Breaks Classification Method

Strata by Jenk's	N	Mean	Variance	Coeff of Variation	Std Error
0	676	0.00	0.00	.	0.00

1	80	25.36	225.26	59.18	1.68

2	81	85.74	390.56	23.05	2.20

3	51	153.43	539.90	15.14	3.25

4	40	248.44	923.50	12.23	4.80

5	28	465.04	31, 521.72	38.18	33.55

For each design alternative the initial number of field plots was set to n = 20, except for stratified sampling, where a minimum of 40 field plots was predefined to obtain a sufficient within-strata sample size. A maximum sample of n = 6, 000 was sufficient to show the effect of increasing sample size on the percent standard error. We sampled n = 20 to 6, 000 in increments of 5.

Costs are decisive for the selection of the optimal design alternative, but are for the most part neglected in publications on inventory concepts for REDD. Reports on costs of different components of an inventory such as ground survey, analysis of remote sensing data, or data cost vary widely. As we did not want to optimize an inventory design for a specific application but illustrate the effect of cost implications on the design selection, we choose a range of realistic costs for field assessments and remote sensing data acquisition and interpretation. Fixed cost components such as administration, training or infrastructure were not included as they are not design dependent. Table 5 shows the costs used in the simulation study. For remote sensing imagery two alternative cost scenarios were utilized. Alternative 1 was chosen according to Asner et al. [41], who quantified the cost for the acquisition of LiDAR data with 0.16 US$/ha for carbon mapping on the Island of Hawaii. Alternative 2 reflects the cost of multispectral imagery, as specified by Häussler (cited in [25]).

**Table 5:** Cost figures used in the simulation study

Cost component	Cost	
	
	**Min**.	**Max**.
Field assessment	500 US$/plot	5, 000 US$/plot

Remote sensing imagery, Alternative 1	0.1 US$/ha	1 US$/ha

Remote sensing imagery, Alternative 2	0.01 US$/ha	-

The following settings of the simulation study were realized:

Field sample size:   n = {20, ... 6, 000}

Remote sensing coverage:   1%, 10%, full

Cost per field plot:   500 US$, 5, 000 US$

Cost remote sensing imagery:   0.1 US$/ha, 1 US$/ha

r^2^-value, AGB = f(remote sensing signal):   0.3, 0.6, 0.9

Sampling designs:   simple random sampling, stratified sampling, regression sampling, 2-phase sampling with regression estimators

Based on the coefficients of variation for the sampling population (Table 3) and the stratification rules presented in Table 4 we calculated the cost-efficiency in terms of total cost and percent standard error or each combination of settings. The simulation was run under SAS^© ^[42].

## Competing interests

The authors declare that they have no competing interests.

## Authors' contributions

MK drafted the manuscript and carried out the simulation study. MK and AL developed the main simulation framework and finalized the manuscript. AL directed MK's disorganized thoughts, made the FIA data available for the study and applied the Jenk's Natural Breaks Classification. CS, TB, and DP contributed to the sampling design selection, provided information on the cost framework, and contributed to the manuscript. All authors read and approved the final manuscript.
